# Multi-Sensor Collaborative Positioning in Range-Only Single-Beacon Systems: A Differential Chan–Gauss–Newton Algorithm with Sequential Data Fusion

**DOI:** 10.3390/s25082577

**Published:** 2025-04-18

**Authors:** Yun Ye, Hongyang He, Enfan Lin, Hongqiong Tang

**Affiliations:** 1 School of Electrical Engineering, Naval University of Engineering, Wuhan 430033, China; m23385406@nue.edu.cn (Y.Y.); hqtang@foxmail.com (H.T.); 2 Department of Mechanics and Engineering Science, College of Engineering, Peking University, Beijing 100091, China; nfun-lin@outlook.com

**Keywords:** underwater navigation, multiple sensors, underwater virtual beacon, Gauss–Newton algorithm

## Abstract

The development of underwater high-precision navigation technology is of great significance for the application of autonomous underwater vehicles (AUVs). Traditional long baseline (LBL) positioning systems require pre-deployment and the calibration of multiple beacons, which consumes valuable time and manpower. In contrast, the range-only single-beacon (ROSB) positioning technology can help autonomous underwater vehicles (AUVs) obtain accurate position information by deploying only one beacon. This method greatly reduces the time and workload of deploying beacons, showing high application potential and cost ratio. Given the operational constraints of AUV open-ocean navigation with single-beacon weak observations and absence of valid a priori positioning data in calibration zones, a multi-sensor underwater virtual beacon localization framework was established, proposing a differential Chan–Gauss–Newton (DCGN) methodology for submerged vehicles. Based on inertial navigation, the method uses the distance measurement information from a single beacon and observations from multiple sensors, such as the Doppler velocity log (DVL) and pressure sensor, to obtain accurate position estimates by discriminating the initial position of multiple hypotheses. A simulation experiment and lake test show that the proposed method not only significantly improves the positioning accuracy and convergence speed, but also shows high reliability.

## 1. Introduction

With the continuous development and utilization of Marine resources, as well as the continuous progress of underwater vehicle technology and Marine scientific research, underwater acoustic navigation technology occupies a core position in the accurate positioning and navigation of autonomous underwater vehicles (AUVs) [1,2]. Given the unique ability of acoustic signals to travel long distances in underwater environments, researchers have developed a variety of positioning techniques. Among them, long-baseline (LBL), short-baseline (SBL), and ultra-short baseline (USBL) underwater acoustic positioning systems have become mainstream solutions [3,4,5]. The LBL system is widely used for prolonged and long-distance missions due to its excellent positioning accuracy and wide operation coverage. However, the pre-deployment and fine calibration requirements of multibeacons is a time-consuming and labor-intensive process [6]. To response this challenge of LBL, a range-only single-beacon (ROSB) technology is come into being, bringing innovative changes to underwater navigation technology [7]. Compared to LBL, the use of ROSB has greatly reduced the consumption of time and labor. The simplification of the system architecture not only reduces the complexity of equipment deployment and maintenance, but also greatly improves the efficiency and flexibility of operations by eliminating the dependence on multibeacons.

In recent years, ROSB has become a research focus due to its ability to accurately position and continuously track AUV by relying only one beacon [8,9]. In the early research, Scherbatyuk [10] initially laid the foundation of underwater single-beacon navigation. Subsequently, a variety of single-beacon assisted positioning methods were further studied, and the application scenarios were constantly expanded. Aditya et al. [11,12] conducted an observability analysis for single-beacon ranging and positioning, which had a profound impact on subsequent studies. In order to meet the challenge of mobile single-beacon positioning, Vaganay and Webster et al. designed least squares (LS) [13] and centralized Extended Kalman filter (CEKF) algorithms [14]. Larsen et al. [15] proposed a comprehensive LBL algorithm combined with surface ship navigation estimation to achieve submeter-level accuracy, and analyzed the observability of system state under specific trajectories. Aiming at the problem of ROSB under the condition of direct route, a path planning method based on observable measure was proposed in the literature [16]. The INS/APS passive single-beacon navigation method proposed by Zou et al. further improves the positioning accuracy of single beacons by applying passive acoustic detection technology [17]. In view of the limitation of initial ROSB observability, Zhao et al. [18] proposed a multi-sensor fusion location method based on factor maps. This innovative approach integrates ROSB with other sensors on AUV to achieve significant positioning accuracy through multi-sensor fusion.

With the continuous development and improvement of single-beacon navigation technology, underwater positioning ability has been promoted significantly. However, ROSB still faces two core challenges: low initial observation capability and limited positioning accuracy. To solve these problems, some of the sensors installed on the AUV, including DVL, Pressure Sensor (PS), and Conductivity Temperature Depth (CTD), can be fused with the position observation information of the ROSB to achieve the estimation and correction of the AUV position. Underwater virtual single-beacon positioning technology is the fusion technology of underwater multi-sensor and ROSB information. By using the velocity information of AUV measured by DVL and the data of ocean CTD sensor, the virtual acoustic beacon array based on multiple ranging information is successfully constructed. By integrating AUV dead reckoning data and single-beacon ranging data, the method simulates the effect of multibeacon positioning, significantly shortens the beacon deployment cycle, and effectively reduces the operational burden. This technology shows a high application potential and cost–benefit ratio, and provides a new idea for the development of underwater navigation and positioning technology.

As early as 2002, Larsen et al. [15] proposed the concept of the virtual beacon and applied a Kalman filter to correct the position. Subsequently, Lapointe et al. [19] deeply discussed the concept of a virtual long baseline (VLBL) in their dissertation, and laid a solid foundation for subsequent research through a detailed experimental analysis. Reference [20] designed an INS/Time Difference of Arrival (TDOA) loosely coupled integrated navigation algorithm. The algorithm uses the inertial navigation position to construct a virtual array, receives TDOA information to solve the source position closed solution, and realizes the inertial navigation position correction. In addition, Yu Yanting et al. [21] systematically described the single-beacon underwater acoustic location method based on VLBL technology, and comprehensively summarized the research progress in this field. While single-beacon positioning systems have achieved substantial research advancements, their operational efficacy faces fundamental constraints in extended-duration operations and long-range navigation scenarios. [22]. Using the traditional least square [23] and Kalman filtering algorithms [24,25] makes it difficult to ensure the reliability and accuracy of the results when dealing with complex positioning problems, especially when the measurement information is insufficient. In contrast, the differential Chan–Gauss–Newton (DCGN) virtual beacon algorithm proposed in this paper is an effective means to solve this problem because of its low computation and high precision without setting the initial value.

Based on the TDOA principle, the Chan algorithm is especially suitable for the case when there are sufficient base stations and the TDOA error conforms to Gaussian distribution. However, the traditional TDOA method is often limited in dealing with nonlinear hyperbolic equations because of the high computational cost and limited precision of nonlinear iteration. To solve these problems, Hao et al. [26] proposed an enhanced AUV-aided TDOA localization algorithm specifically for underwater acoustic sensor networks, which achieves high accuracy while reducing computational complexity and effectively overcomes the shortcomings of the Chan algorithm. In addition, in order to deal with the impact of low signal-to-noise ratio (SNR) and reverberation environment on positioning accuracy, an EMD-ML hybrid method is proposed, which further improves the overall accuracy of positioning system by restricting the error of the Chan algorithm [27]. It is worth noting that the efficiency of nonlinear iterative algorithms greatly depends on the accuracy of initial values, and inaccurate initial values can easily lead to algorithm divergence. Especially under the influence of large range error and non-line-of-sight (NLOS) error, the performance of the Chan algorithm will obviously decrease. To solve the problem that AUVs are susceptible to NLOS errors and noise deviation from the receiving station, Yang, H et al. [28] proposed a weighted modified Chan–Taylor (WMChan–Taylor) algorithm. By introducing dynamic weights to adjust the noise variance of the measuring station, the positioning accuracy of the AUV is significantly improved. The Chan–Taylor co-location algorithm proposed by Hua et al. [29] aims to reduce errors, but Taylor algorithm has problems such as sensitivity to initial value, data redundancy and high computational complexity, which need to be taken into account in practical applications. The Gauss–Newton iterative method, as an efficient optimization tool for nonlinear least squares problems [30], cleverly combines the fast convergence characteristic of the Gauss–Newton method with the flexibility of the iterative method. It linearizes the complex equation by performing the Taylor series expansion near the solution of the equation, estimates the deviation between the initial value and the true value by using the least square method, and iterates continuously until the preset accuracy requirements are met. In order to improve its convergence, Q. H. Liu [31] designed a relaxation Gauss–Newton (RGN) method by introducing relaxation factors, and verified its improved convergence through numerical experiments. Although the Gauss–Newton iteration method can ensure the accuracy of the solution after many iterations, the effectiveness of the method is highly dependent on the initial value close to the true value [32]; otherwise, it may fall into a local optimum. To solve this limitation, Liu et al. [31] proposed an innovative Gauss–Newton optimal step size strategy, which significantly improved the estimation accuracy of positioning iteration.

Underwater acoustic signals are highly susceptible to interference, making it challenging for hydrophone arrays to simultaneously maintain the stable reception of time-delay data from four channels. Furthermore, there is no guarantee that the acquired time-delay signals meet the precision requirements for positioning. When AUVs navigating in the open ocean enter calibration zones, their Strapdown Inertial Navigation Systems (SINSs) typically accumulate positioning errors spanning several nautical miles. Without reliable a priori position information, positional calibration becomes unfeasible. Consequently, it is imperative to investigate ROSB localization methodologies. The Chan algorithm is affected by the ranging error, and the Gauss–Newton algorithm is sensitive to the initial value, so this paper innovatively proposes a DCGN localization algorithm based on ROSB to realize underwater the high-precision positioning of underwater vehicles. In order to save the time and manpower of beacon deployment, this algorithm first establishes a virtual beacon positioning model based on AUV kinematics information and virtual beacon ranging information. Then, the Chan algorithm is used to quickly obtain the preliminary solution that meets the iterative conditions. The improved Gauss–Newton algorithm is used as the input for iterative optimization to obtain higher-precision positioning results. Furthermore, a DCGN/SINS integrated navigation scheme is proposed to solve the problem of the invalidity of long-endurance information of the single beacon. The proposed DCGN algorithm, based on ROSB, is combined with an SINS to achieve the accurate calibration of AUVs by the on-line estimation and compensation of device errors. This approach ensures positional calibration capability even in the complete absence of prior valid location data upon entering calibration zones, while enabling short-term navigation and positioning. Such a solution addresses SINS calibration challenges for AUVs with minimal operational overhead.

## 2. Motion Parameter Transfer and Framework of Virtual Beacon

### 2.1. Motion Parameter Transfer Analysis

To accurately obtain the pitch, roll, and heading angles of the carrier, “right-front-upward” was selected as the body frame (b-frame). To realize global inertial navigation and positioning, the geocentric inertial system (i-frame) is introduced. The Earth-Centered Earth-Fixed (ECEF) coordinate system is a rigidly attached reference frame rotating synchronously with the Earth’s surface. The output and calculation of navigation parameters in a strapdown inertial navigation system are performed in the navigation coordinate system. Therefore, the “East-North-Up” (E-N-U) geographic coordinate system is selected as the navigation reference coordinate system (n-frame) to describe the attitude, velocity, and position update algorithms. The transformation between ECEF coordinates $p^{e}=(x,y,z)$ and geographic coordinates $p^{n}=(L,\lambda ,h)$ (latitude, longitude, and altitude) is defined as follows:

(1) $$\left\{\begin{array}{l} x=\left(R_{N}+h\right)\cos L\cos\lambda \\ y=\left(R_{N}+h\right)\cos L\sin\lambda \\ z=\left[R_{N}\left(1-e^{2}\right)+h\right]\sin L \end{array}\right.$$

where $R_{M}=R_{e}(1-e^{2})/{\left(1-e^{2}sin^{2}L\right)}^{3/2}$ and $R_{N}=R_{e}/{\left(1-e^{2}sin^{2}L\right)}^{1/2}$ represent the meridian and prime vertical radii of curvature, $R_{e}=6378137$ is the Earth’s semi-major axis, and e=0.0818 denotes the reference ellipsoid eccentricity.

The attitude and speed errors of inertial navigation are periodic, so it is ideal to use the speed of the log when the ranging period is long or the carrier is far away from the beacon. The log can measure the speed under the system more accurately after calibration, and the speed conversion relationship between the system is as follows:

(2) $$v^{n}=[v_{E}^{n},v_{N}^{n},v_{U}^{n}]=C_{b}^{n}v^{b}$$

where velocity components $v_{E}$, $v_{N}$, $v_{U}$ correspond to east, north, and up directions and $C_{b}^{n}$ represents the attitude rotation matrix of the b-frame with respect to the n-frame. By converting the geographical coordinates before and after the ranging period interval to the geocentric cartesian coordinates, the carrier motion parameters can be obtained and calculated in the geocentric cartesian coordinate system.

### 2.2. Framework of Virtual Beacon Positioning

**Figure 1:**
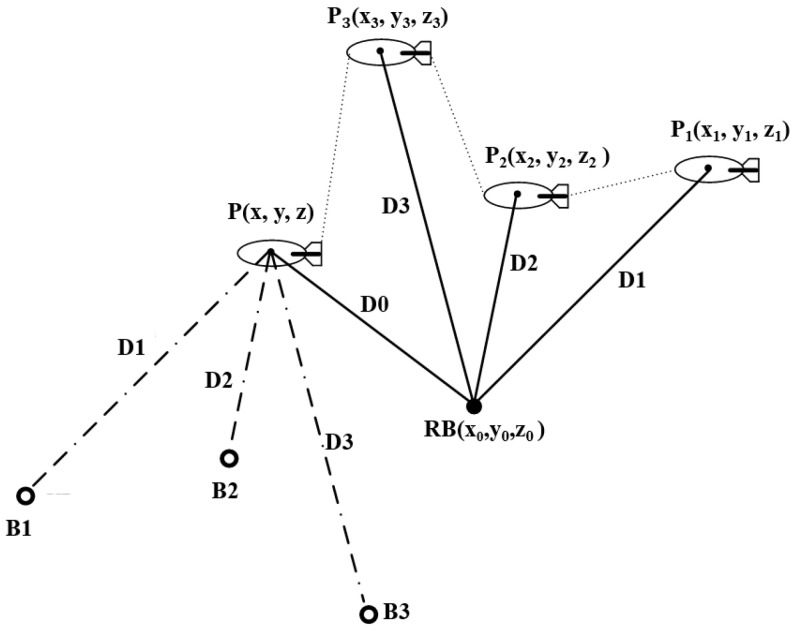
Schematic diagram of the virtual beacon’s operational principle.

In the field of underwater acoustic location, the spherical position information brought by a single distance measurement increases the uncertainty of location. In order to overcome this limitation, a virtual acoustic beacon array is constructed by combining the short-time high-precision characteristic of the inertial navigation system and using multiple ranging strategies to accumulate data [18,21]. The construction principle of the virtual beacon is shown in Figure 1.

The positioning system comprises a single acoustic beacon deployed on the seafloor and a transponder mounted on the underside of an underwater vehicle. The seafloor-deployed beacon’s position coordinates are precisely known, with the acoustic source continuously emitting periodic acoustic signals. This configuration enables underwater navigation through Time-of-Arrival (TOA) measurements and geometric triangulation principles [33]. After the AUV enters the array element area, it makes a sound to wake up the submarine array and synchronizes the time, and the submarine array emits the acoustic signal periodically. Therefore, in a period, if the AUV receives the signal at time $t_{i}$ and the array element sends out the signal at time $t_{0}$, the propagation time of the sound signal in water is $\Delta t_{i}=t_{i}-t_{0}$, and the distance between the AUV and the array element is $D_{i}=\bar{c}\cdot \Delta t_{i}$, where $\bar{c}$ is the equivalent average sound velocity, which is obtained from the sound velocity profile.

The navigation parameter output and computation of the SINS are performed in the n-frame. Similarly, the positioning provided by acoustic beacons is also referenced to the n-frame. During prolonged ranging cycles or when the AUV operates at significant distances from the beacon, the calibrated DVL can accurately measure velocity in the b-frame. The velocity transformation relationship between the b-frame and n-frame can be rigorously expressed by Equation (2).

The RB positioning coordinates are a priori known, and AUV position information is obtained by pure inertial navigation. Assuming the geographic coordinates $RB^{n}=(L,\lambda ,h)$ (longitude, latitude, altitude) of an underwater acoustic beacon are transformed via Equation (1), the resulting ECEF coordinates are denoted as $RB={\left[x_{0},y_{0},z_{0}\right]}^{T}$. $P_{1}$ is the initial state of AUV, and $P_{2}$, $P_{3}$, and P represent states in the following three slots. The position coordinates corresponding to the AUV from $P_{1}$ to P is $P_{i}={\left(x_{i},y_{i},z_{i}\right)}^{T},i=1,2,3,4$, and the ranging value obtained from the corresponding position $P_{1}$ to P is $D_{1}$, $D_{2}$, $D_{3}$, and $D_{0}$. According to the geometric position relationship in Figure 1, the equation can be listed as follows:

(3) $$\left\{\begin{array}{l} {\left(x_{0}-x_{1}\right)}^{2}+{\left(y_{0}-y_{1}\right)}^{2}+{\left(z_{0}-z_{1}\right)}^{2}=D_{1}^{2}\\ {\left(x_{0}-x_{2}\right)}^{2}+{\left(y_{0}-y_{2}\right)}^{2}+{\left(z_{0}-z_{2}\right)}^{2}=D_{2}^{2}\\ {\left(x_{0}-x_{3}\right)}^{2}+{\left(y_{0}-y_{3}\right)}^{2}+{\left(z_{0}-z_{3}\right)}^{2}=D_{3}^{2}\\ {\left(x_{0}-x\;\right)}^{2}+{\left(y_{0}-y\;\right)}^{2}+{\left(z_{0}-z\;\right)}^{2}=D_{0}^{2} \end{array}\right.$$

The relationship between the position of an AUV undergoing four ranging cycles is $\Delta P_{i}=P_{0}-P_{i},i=1,2,3$, and $P={\left(x,y,z\right)}^{T}$ is regarded as the position to be solved, and $B_{i}={\left[x_{0}+\Delta x_{i},y_{0}+\Delta y_{i},z_{0}+\Delta z_{i}\right]}^{T}$(i=1,2,3), such that

(4) $$\left\{\begin{array}{l} {\left(x_{B1}-x_{4}\right)}^{2}+{\left(y_{B1}-y_{4}\right)}^{2}+{\left(z_{B1}-z_{4}\right)}^{2}=D_{1}^{2}\\ {\left(x_{B2}-x_{4}\right)}^{2}+{\left(y_{B2}-y_{4}\right)}^{2}+{\left(z_{B2}-z_{4}\right)}^{2}=D_{2}^{2}\\ {\left(x_{B3}-x_{4}\right)}^{2}+{\left(y_{B3}-y_{4}\right)}^{2}+{\left(z_{B3}-z_{4}\right)}^{2}=D_{3}^{2}\\ {\left(x_{RB}-x\;\right)}^{2}+{\left(y_{RB}-y\;\right)}^{2}+{\left(z_{RB}-z\;\right)}^{2}=D_{0}^{2} \end{array}\right.$$

$B_{1}$ is the virtual beacon obtained by the AUV at the position of $P_{1}$ and two virtual beacons $B_{2}$ and $B_{3}$ are obtained in the same way. It is equivalent to transferring the motion parameters of the underwater carrier to the actual acoustic beacon, and then constructing three virtual beacons which together form $RB={\left[x_{0},y_{0},z_{0}\right]}^{T}$, a new virtual beacon array to solve the position of P.

## 3. Basic Principle of Positioning Algorithm

### 3.1. Chan Algorithm

The Chan algorithm is a location algorithm based on TDOA [29]. The algorithm’s fundamental mechanism measures the TDOA-derived distance disparity between the primary station and auxiliary stations, subsequently employing hyperbolic equations formulated from base station coordinates to resolve target localization [34,35].

**Figure 2:**
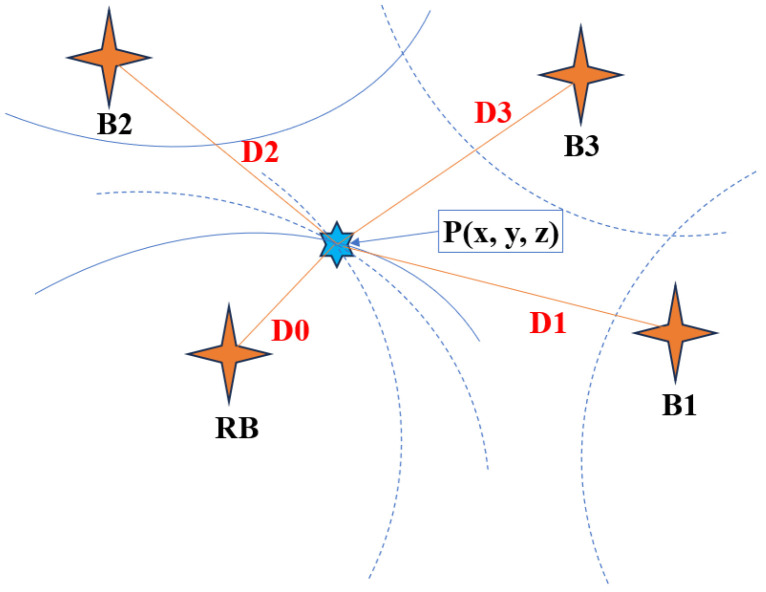
Chan algorithm positioning model diagram.

In Figure 2, $B_{1}$, $B_{2}$, $B_{3}$, and RB represent the known receiving base station. The coordinates of B at the *i*-th position are $\left[x_{i},y_{i},z_{i}\right]$$\left(i=1,2,3\right)$, three hyperbolas are drawn with $RB={\left[x_{0},y_{0},z_{0}\right]}^{T}$ as the basis, the intersection point of the three hyperbolas is P (to be positioned), the coordinate is set as (x,y,z), the distance between P and $B_{i}$ is $D_{i}$, and the distance between P and RB is $D_{0}$. Based on the above analysis, the distance equation is obtained:

(5) $$D_{i}^{2}={\left(x_{i}-x\right)}^{2}+{\left(y_{i}-y\right)}^{2}+{\left(z_{i}-z\right)}^{2}=K_{i}-2x_{i}x-2y_{i}y-2z_{i}z+D\;(i=0,1,2,3)$$

where $K_{i}=x_{i}^{2}+y_{i}^{2}+z_{i}^{2}$, $D=x^{2}+y^{2}+z^{2}$. Let $D_{j}-D_{i}=D_{i,j}$ represent the difference between the distance from $B_{j}$ to P (RB to P) and the distance from $B_{i}$ to P, then $D_{i}^{2}=D_{i,j}^{2}+D_{j}^{2}+2D_{i,j}D_{j}$.

Equation (5) is subtracted from the distance equation of RB, and sorted into the form of linear equations.

(6) $$\left[\begin{array}{lll} x_{1}-x_{0} & y_{1}-y_{0} & z_{1}-z_{0}\\ x_{2}-x_{0} & y_{2}-y_{0} & z_{2}-z_{0}\\ x_{3}-x_{0} & y_{3}-y_{0} & z_{3}-z_{0} \end{array}\right]\left[\begin{array}{c} x\\ y\\ z \end{array}\right]=\frac{1}{2}\left(\begin{array}{l} K_{1}-K_{0}-D_{0,1}^{2}\\ K_{2}-K_{0}-D_{0,2}^{2}\\ K_{3}-K_{0}-D_{0,3}^{2} \end{array}\right)-\left(\begin{array}{l} D_{0,1}\\ D_{0,2}\\ D_{0,3} \end{array}\right)D_{0}$$

The position of the solvable tag is $X_{P\;}=A^{-1}b$, where

(7) $$\begin{array}{l} A=\left[\begin{array}{lll} x_{1}-x_{0} & y_{1}-y_{0} & z_{1}-z_{0}\\ x_{2}-x_{0} & y_{2}-y_{0} & z_{2}-z_{0}\\ x_{3}-x_{0} & y_{3}-y_{0} & z_{3}-z_{0} \end{array}\right]\\ b=\frac{1}{2}\left(\begin{array}{l} K_{1}-K_{0}-D_{0,1}^{2}\\ K_{2}-K_{0}-D_{0,2}^{2}\\ K_{3}-K_{0}-D_{0,3}^{2} \end{array}\right)-\left(\begin{array}{l} D_{0,1}\\ D_{0,2}\\ D_{0,3} \end{array}\right)D_{0} \end{array}.$$

### 3.2. Gauss–Newton Algorithm

The Gauss–Newton iteration algorithm serves as the pivotal solution for overcoming singular Jacobian conditions in nonlinear equations that may lead to unsolvable states, demonstrating superior capability in performing the nonlinear optimization of positioning equations [36]. The Gauss–Newton iterative method operates by linearizing nonlinear residual functions through Taylor series expansions (while neglecting higher-order terms), then iteratively refining parameter estimates by solving successive linear least-squares approximations until convergence to the minimal residual sum of squares is achieved [37]. This not only improves the computational efficiency but also effectively deals with complex nonlinear relationships, making the regression model more accurate and reliable [38].

The coordinate of RB is ${\left[x_{0},y_{0},z_{0}\right]}^{T}$, given n points where $B_{i}={\left[x_{0}+\Delta x_{i},y_{0}+\Delta y_{i},z_{0}+\Delta z_{i}\right]}^{T}={\left[x_{i},y_{i},z_{i}\right]}^{T}(i=1,2\cdots n)$, respectively, and $P={\left(x,y,z\right)}^{T}$ represents the coordinates of the points to be found. The distance between RB and P is $D_{0}$, and the distance between point P and $B_{i}$ is $D_{i}$(i=1,2⋯n). There is a distance relationship as follows:

(8) $$D_{i}=\sqrt{{\left(x-x_{i}\right)}^{2}+{\left(y-y_{i}\right)}^{2}+{\left(z-z_{i}\right)}^{2}}$$

Equation (8) can be written as a multivariate function with coordinates of P=(x,y,z).

(9) $$f(x,y,z)=\sqrt{{\left(x-x_{i}\right)}^{2}+{\left(y-y_{i}\right)}^{2}+{\left(z-z_{i}\right)}^{2}}$$

$P_{c}={\left(x_{c},y_{c},z_{c}\right)}^{T}$ is obtained by the Chan algorithm near the true position or inertial navigation-indicated position (within the iterative threshold range), and the relationship between P and $P_{c}$ is $P={\left(x,y,z\right)}^{T}={\left(x_{c}+\Delta x,y_{c}+\Delta y,z_{c}+\Delta z\right)}^{T}$. Equation (9) performs the Taylor expansion at P and omits the higher-order remainder to obtain Equation (10).

(10) $$f\left(x_{c},y_{c},z_{c}\right)=D_{ic}+\frac{x_{c}-x_{i}}{D_{ic}}\Delta x+\frac{y_{c}-y_{i}}{D_{ic}}\Delta y+\frac{z_{c}-z_{i}}{D_{ic}}\Delta z,$$

In (10), $D_{ic}=\sqrt{{\left(x_{c}-x_{i}\right)}^{2}+{\left(y_{c}-y_{i}\right)}^{2}+{\left(z_{c}-z_{i}\right)}^{2}}(i=0,1,2\cdots n)$, and substituting (10) into (8) can be written as a linear system of the form AX=b, as shown in (11).

(11) $$\left[\begin{array}{ccc} \left(x_{c}-x_{0}\right)/D_{0c} & \left(y_{c}-y_{0}\right)/D_{0c} & \left(z_{c}-z_{0}\right)/D_{0c}\\ \vdots & \vdots & \vdots \\ \left(x_{c}-x_{n}\right)/D_{nc} & \left(y_{c}-y_{n}\right)/D_{nc} & \left(z_{c}-z_{n}\right)/D_{nc} \end{array}\right]\left[\begin{array}{c} \Delta x\\ \Delta y\\ \Delta z \end{array}\right]=\left[\begin{array}{c} D_{0}-D_{0c}\\ \vdots \\ D_{n}-D_{nc} \end{array}\right]$$

When n=2, the matrix is of full rank and the solution is $X=A^{-1}b={\left(\Delta x,\Delta y,\Delta z\right)}^{T}$; when n>2, (11) is the overdetermined system of equations, and $X={\left(A^{T}A\right)}^{-1}\left(A^{T}b\right)$ is the least squares solution. When the condition ${\left\|X\right\|}_{2}\leq \varepsilon$ (L2-norm) is met [39], the iteration is stopped, and the value of ε is one order of magnitude higher than the ranging accuracy according to [40]. If the condition is not met, $P_{c}={\left(x_{c}+\Delta x,y_{c}+\Delta y,z_{c}+\Delta z\right)}^{T}$ is used, and the iteration is carried out again until the condition is met.

## 4. DCGN Positioning Method with Underwater Virtual Beacon

**Figure 3:**
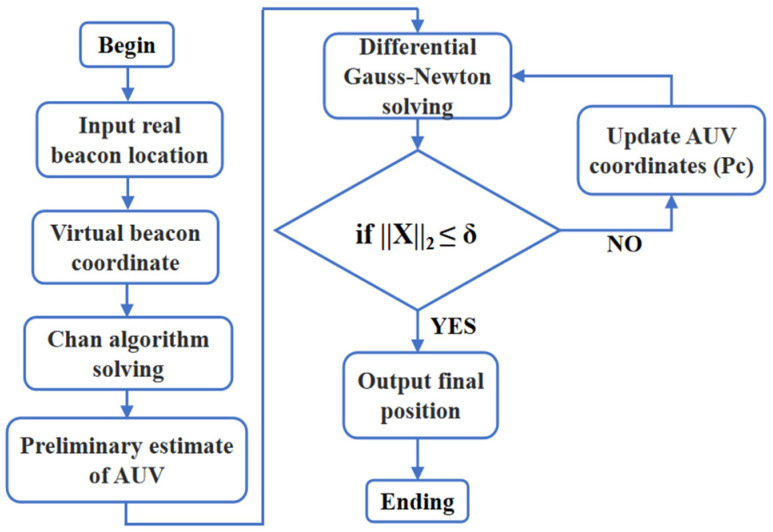
DCGN virtual beacon positioning algorithm flowchart.

Underwater vehicles often enter the target beacon area with significant positioning errors. Due to the complexity of the underwater environment and susceptibility of acoustic signal propagation to interference, these vehicles struggle to simultaneously receive signals from multiple beacons or ensure high-quality signals across multiple array elements of their acoustic arrays. This makes it difficult to guarantee initial iteration values near the true values for the first step of the algorithm. To address this, this paper proposes a hybrid method leveraging the complementary strengths of the Chan algorithm and the Gauss–Newton iterative algorithm. Specifically, the Chan algorithm first generates a solution meeting iteration criteria, which is then refined through the differential Gauss–Newton algorithm to achieve high-precision positioning. Together, these form a virtual beacon-based localization framework, as shown in Figure 3.

**Figure 4:**
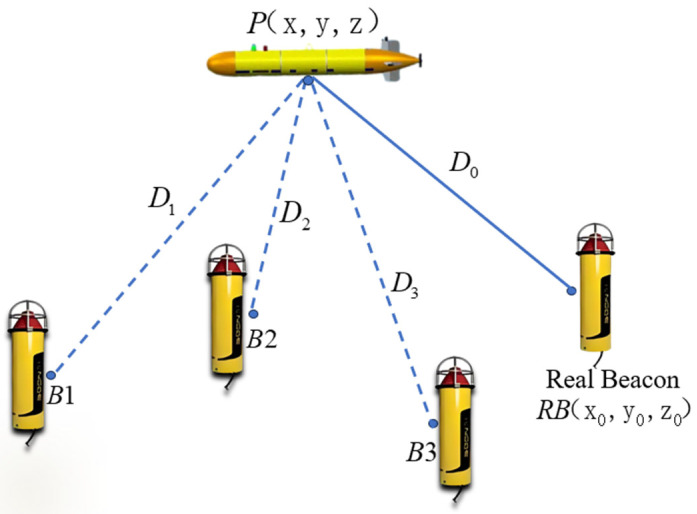
Reference array element and valid element.

In the Figure 4 for constructing virtual beacon array, a matrix system containing n + 1 number is set up, and the real beacon RB ($RB={\left[x_{0},y_{0},z_{0}\right]}^{T}$) is selected as the reference matrix. According to the above derivation, it can be seen that other effective primitive elements are $B_{i}={\left[x_{0}+\Delta x_{ni},y_{0}+\Delta y_{ni},z_{0}+\Delta z_{ni}\right]}^{T}=\left[x_{i},y_{i},z_{i}\right](i=1,2\cdots n)$. The distance between $B_{i}$ and the AUV is $D_{i}(i=1,2\cdots n)$, the distance between RB and the AUV is $D_{0}$. The coordinate of the point to be obtained for the AUV is $P={\left(x,y,z\right)}^{T}$.

According to the real-time kinematic in satellite navigation, the partial error sources carried by multiple distance ranging signals near the same acoustic beacon are basically the same. The ranging equation is (12).

(12) $$D_{i}=\sqrt{{\left(x-x_{i}\right)}^{2}+{\left(y-y_{i}\right)}^{2}+{\left(z-z_{i}\right)}^{2}},$$

where i=0,1,2⋯n, and when i=0, Equation (12) is the ranging equation of RB. The difference between $D_{0}$ and $D_{i}$ is written as a multivariate function of the underwater carrier point coordinate P to obtain (13).

(13) $$f(x,y,z)=\sqrt{{\left(x-x_{0}\right)}^{2}+{\left(y-y_{0}\right)}^{2}+{\left(z-z_{0}\right)}^{2}}-\sqrt{{\left(x-x_{i}\right)}^{2}+{\left(y-y_{i}\right)}^{2}+{\left(z-z_{i}\right)}^{2}}$$

The real position of the point coordinate to be obtained is $P={\left(x,y,z\right)}^{T}$, so let $X_{ci}=\frac{x_{c}-x_{i}}{D_{ic}},\;Y_{ci}=\frac{y_{c}-y_{i}}{D_{ic}},\;Z_{ci}=\frac{z_{c}-z_{i}}{D_{ic}},D_{ic}=\sqrt{{\left(x_{c}-x_{i}\right)}^{2}+{\left(y_{c}-y_{i}\right)}^{2}+{\left(z_{c}-z_{i}\right)}^{2}},(i=0,1,2\cdots n)$ and $P_{c}={\left(x_{c},y_{c},z_{c}\right)}^{T}$ is obtained by the Chan algorithm near the true position or inertial navigation-indicated position (within the iterative threshold range). It can be readily demonstrated that f(x,y,z) is continuous and has an order continuous partial derivative in a certain domain of $P_{c}={\left(x_{c},y_{c},z_{c}\right)}^{T}$. Then, the Taylor series is expanded at $P_{c}$ to linearize (13), and the higher-order terms are omitted to obtain Equation (14).

(14) $$f\left(x_{c},y_{c},z_{c}\right)=D_{0c}-D_{ic}+\left(X_{c0}-X_{ci}\right)\Delta x+\left(Y_{c0}-Y_{ci}\right)\Delta y+\left(Z_{c0}-Z_{ci}\right)\Delta z$$

Equation (14) is written as a system of linear equations of the form of (15).

(15) AX=b,

where

$$\begin{array}{l} A=\left[\begin{array}{ccc} X_{c0}-\left(x_{c}-x_{1}\right)/D_{1}c & Y_{c0}-\left(y_{c}-y_{0}\right)/D_{1}c & Z_{c0}-\left(z_{c}-z_{0}\right)/D_{1c}\\ \vdots & \vdots & \vdots \\ X_{c0}-\left(x_{c}-x_{n}\right)/D_{n}c & Y_{c0}-\left(y_{c}-y_{n}\right)/D_{n}c & Z_{c0}-\left(z_{c}-z_{n}\right)/D_{nc} \end{array}\right]\\ b=\left[\begin{array}{c} D_{0}-D_{1}-D_{0c}+D_{1c}\\ \vdots \\ D_{0}-D_{n}-D_{0c}+D_{nc} \end{array}\right]\;\;\;\;\;\;\;\;\;\;\;\;\;\;\;X=\left[\begin{array}{c} \Delta x\\ \Delta y\\ \Delta z \end{array}\right] \end{array}.$$

When n=3, the matrix is of full rank, and the solution is $X=A^{-1}b={\left(\Delta x,\Delta y,\Delta z\right)}^{T}$; when n>3, (15) is the overdetermined system of equations, and $X={\left(A^{T}A\right)}^{-1}\left(A^{T}b\right)$ is the least squares solution. When the condition ${\left\|X\right\|}_{2}\leq \varepsilon$ is met, the iteration is stopped, and the value of ε is one order of magnitude higher than the ranging accuracy according to experience. If the condition is not met, $P_{c}={\left(x_{c}+\Delta x,y_{c}+\Delta y,z_{c}+\Delta z\right)}^{T}$ is used, and the iteration is carried out again until the condition is met.

## 5. Simulation Test and Lake Trial Test Research

### 5.1. Simulation Test Analysis

**Figure 5:**
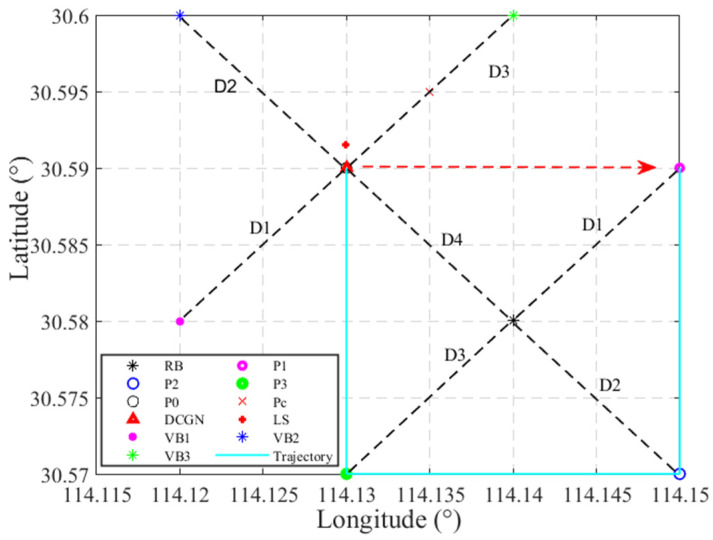
Location model diagram.

The accuracy of the proposed DCGN positioning method based on a virtual beacon is affected by many factors, such as the ranging error, motion parameter transfer error, geometric layout of the virtual beacon, and iteration initial value. The geometric layout of the virtual beacon is particularly serious, which can be effectively solved by the precise control of the underwater carrier track in practical applications. To avoid rank deficiency in the Jacobian matrix of the nonlinear equations, the AUV is required to periodically adjust its depth or heading during navigation. This strategy ensures the observability of the system and prevents numerical instability caused by singular configurations. In the simulation analysis, we made it clear that the velocity measurement accuracy of the program instrument was ±0.05%±5mm/s, and the ranging error was strictly controlled within 1% of the slope distance, while ensuring good geometric layout conditions. In order to show the positioning effect more intuitively in the diagram, we set the error of the initial value of the iteration to 0.7 nautical miles (nmi). On this basis, we used the LS model and DCGN algorithm, respectively, for positioning, and the positioning results are shown in Figure 5.

In Figure 5, the black asterisk marks the geographic location of the underwater beacon at (30.58° N, 114.14° E). In the preset calibration area, the underwater carrier starts from (30.59° N, 114.16° E) and passes through four key ranging points successively. These points are represented by pink, blue, green, and black circles, corresponding to the first, second, third, and fourth ranking points, respectively. The final fixing position coordinate is (30.59° N, 114.13° E). The red dashed-line arrows denote the positioning trajectory.

**Figure 6:**
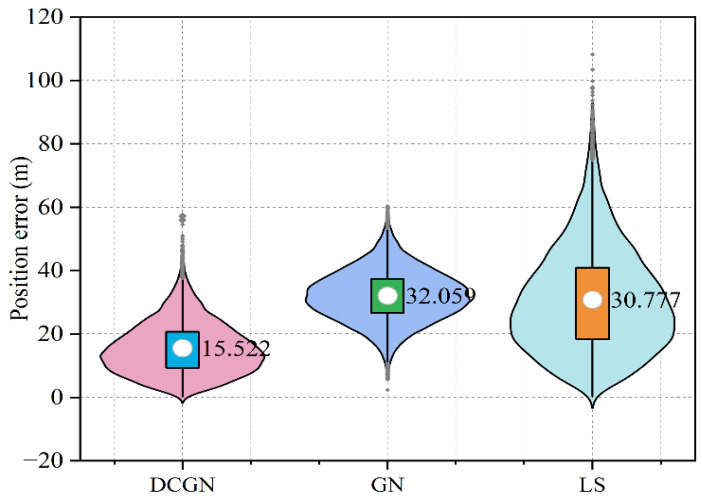
The result of the Monte Carlo simulation.

To verify the stability and reliability of the algorithm, 10,000 Monte Carlo simulations were performed using the LS algorithm, GN algorithm, and DCGN algorithm, respectively. Figure 6 shows the average plane error of the three algorithms in the Monte Carlo simulation under the condition that the initial iterative value is set to conform to the Gaussian error distribution of 0.3 nmi.

The simulation results show that the proposed DCGN algorithm and CGN algorithm have a dense error distribution and small variation range of plane error. However, the error distribution of the LS algorithm is more discrete and has more outliers. In the simulation experiment, the DCGN algorithm can reach the convergence state after an average of five iterations, and the average positioning error is 15.522 m. In contrast, the LS algorithm requires an average of about eight iterations, and its average positioning error is 30.777 m, while the Gauss–Newton algorithm has an average positioning error of 32.059 m.

### 5.2. Lake Trial Test Research

**Figure 7:**
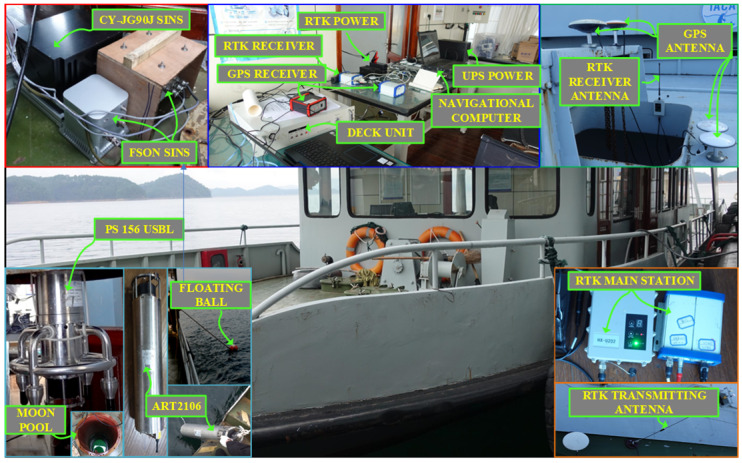
Shipboard test platform.

In order to further verify the feasibility of the proposed method, a test platform based on surface vessels was built in the test site and relevant tests were carried out, as shown in Figure 7. The platform uses high-precision RTK-GNSS and IMU-integrated navigation technology to provide accurate attitude, speed, and position data for the test. In order to realize the real-time synchronization of the test data, the GNSS Pulse Per Second (PPS) signal is used as the benchmark, and the navigation computer generates pulse signals matching the output frequency of each sensor, so as to ensure the time synchronization between each sensor.

The test ship was equipped with two types of strapdown inertial navigation systems: two fiber optic gyro strapdown inertial navigation systems and one laser gyro strapdown inertial navigation system, which are mutually backed up and fixed on the deck of the survey ship. During the test, the data update rate of the inertial navigation system (INS) is 100 Hz, and the position data update rate of the global navigation satellite system (GNSS) is 10 Hz. Table 1 shows the main performance indicators of the optical fiber IMU.

Assuming that the shipborne test platform obtains attitude benchmark $C_{b,refer}^{n}$ and speed benchmark $v_{refer}^{n}$ through the SINS/GNSS integrated navigation system, the output data $v_{DVL}^{b}$ of semi-physical simulation of DVL are shown in Equation (16).

(16) $$v_{DVL}^{b}={\left(C_{b,refer}^{n}\right)}^{T}v_{refer}^{n}$$

Based on the output data $v_{DVL}^{b}$ of DVL as a benchmark, and add ±0.5%±5 cm/s speed measurement errors. The errors have two components: one is the constant shift of ±0.5% for successive starts; another is the random walk of ±5 cm/s. Then, the attitude information of the SINS is used to convert the velocity into the navigation system, and the east-oriented and north-oriented velocity components are obtained. These velocity components are used to transfer motion parameters to construct a virtual beacon array. The entire construction process follows Equation (17).

(17) $$v_{DVL}^{n}=C_{b}^{n}\left(0.005v_{DVL}^{b}+v_{DVL}^{b}+0.05X\right)$$

where $C_{b}^{n}$ is the attitude matrix provided by the SINS at each time. X follows the standard Gaussian distribution $X∼N\left(0,1\right)$ and the data are output at a frequency of 1 Hz.

**Figure 8:**
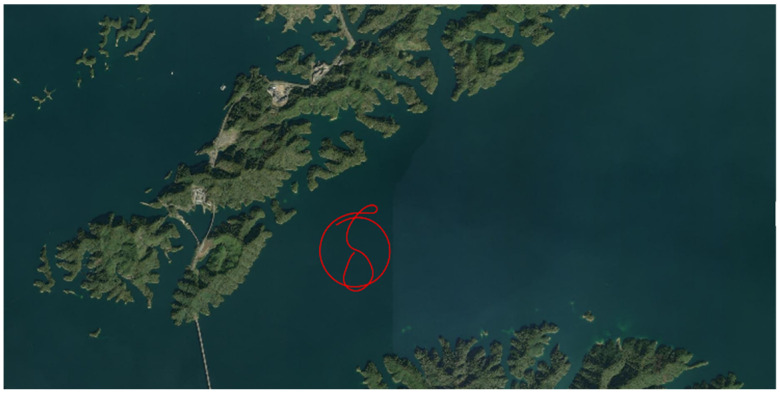
The trajectory of the test ship.

**Figure 9:**
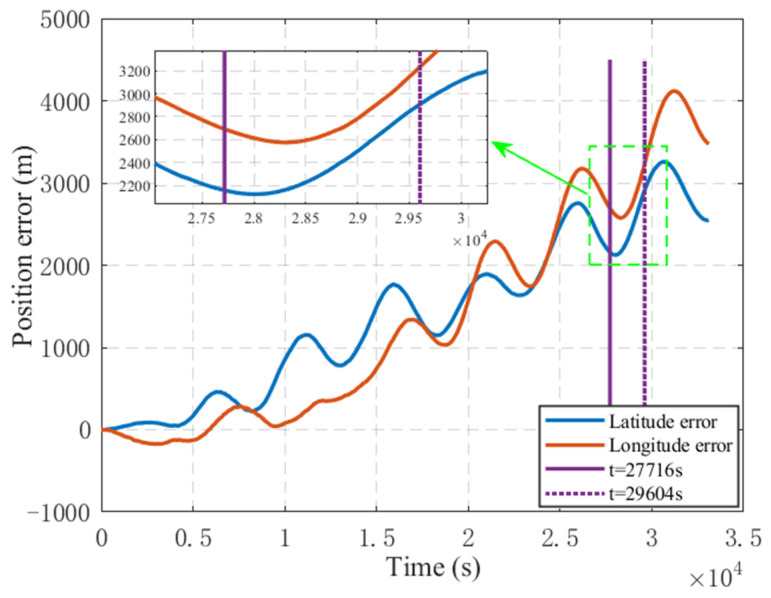
Position error.

In the test, the initial attitude error of inertial navigation is set to 0.1′, the velocity error is 0.01 m/s, and the position error is 1 m. The test ship’s trajectory around the beacon at (29.57° N,118.96° E) is used to verify the method described in this paper. Figure 8 shows the track of the measuring ship. This section of data lasted 472 acoustic positioning cycles, with a cycle interval of 4 s, lasting 1888 s in total. Before entering the standard campus, the SINS had been working for 27,716 s, as shown in Figure 9 Before entering the beacon calibration area, the SINS had accumulated a position error of about 2 nmi.

**Figure 10:**
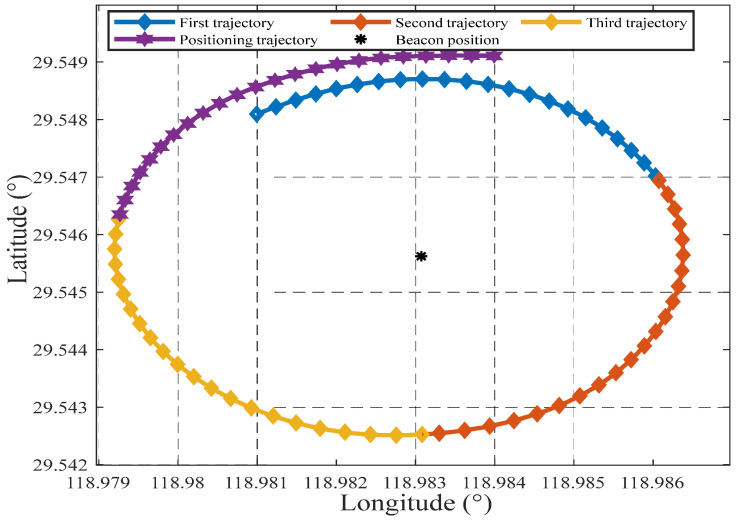
Mark the trajectory within the calibration area.

Figure 10 shows that the overall trajectory in the beacon calibration area took 1040 s to go through 260 ranging cycles. The division of the trajectory follows the following principles: the first 25% of the trajectory is set as the first ranging part, the next 25% is the second ranging part, the next 25% is the third ranging part, and the last 25% is the positioning part. To ensure accurate positioning, the system has stored all sensor information in the first 75% of the trajectory before entering the last 25% of the trajectory.

A novel data processing method is proposed when it is impossible to obtain real-time multi-element delay information to realize location. First, the multi-array time delay information in a given data segment is averaged, and then the equivalent average sound velocity is calculated by combining the sound velocity data measured by the sound velocity profiler, so as to obtain the key information needed for ranging. For the test area, the water depth range is between 40 and 60 m, the depth of the beacon is about 50 m, and the equivalent average speed of sound is about 1473 m/s.

**Figure 11:**
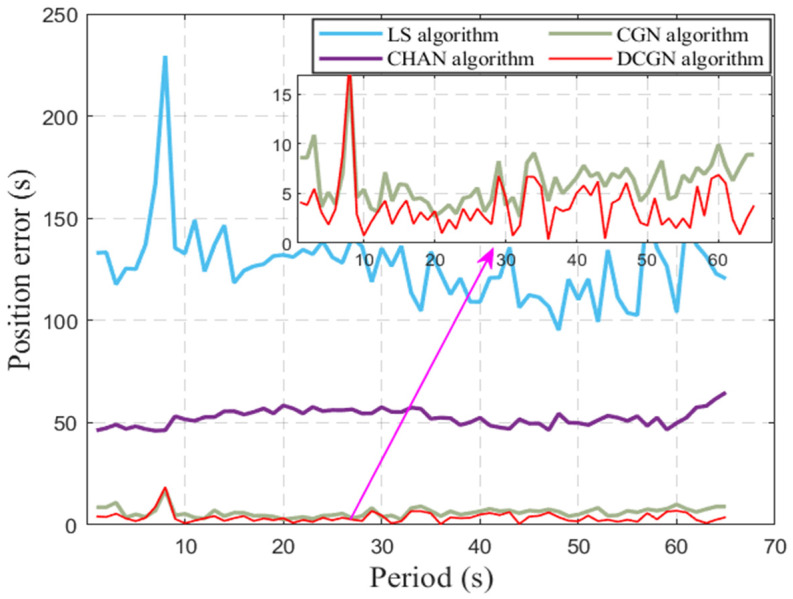
Comparison of position error of four algorithms.

Figure 11 shows the positioning results of the four algorithms. Among them, the blue solid line represents the positioning result of the LS algorithm. Due to the accumulation of 2 nmi of error during the long sea voyage, the average positioning error of LS algorithm is 127.2 m and the standard deviation is 19.01 m. The purple solid line represents the positioning result of the Chan algorithm, whose average positioning error is 52.58 m and standard deviation is 4.06 m. The green solid line shows the CGN algorithm positioning results obtained with the Chan algorithm as the iterative initial value. The average positioning error of CGN is significantly reduced to 6.121 m, and the standard deviation is 2.374 m. Finally, the red solid line reflects the positioning results of the DCGN algorithm, whose average positioning error is further optimized to 3.711 m with a standard deviation of 2.638 m.

In order to calibrate the AUV, the positioning results after DCGN optimization are used as external observation for integrated navigation with the SINS. This study focuses on underwater inertial-based hydroacoustic integrated navigation. Since the moving platform exhibits limited mobility and the inertial navigation system undergoes pre-delivery system calibration, scale factor errors can be neglected. Let the attitude error be denoted as $\varphi \in R^{3}$, velocity error as $\delta v^{n}\in R^{3}$, position error as $\delta p^{n}\in R^{3}$, gyro bias as $\varepsilon^{b}\in R^{3}$, accelerometer bias as $\nabla^{b}\in R^{3}$, and composite error state as $x≜(\varphi ;\delta v^{n};\delta p^{n};\varepsilon^{b};\nabla^{b})\in R^{15}$. The differential equations are formulated as follows:

(18) $$\begin{array}{c} \dot{x}(t)=F(t)x(t)+G(t)w(t)\\ \;\;\;\;\;\;\;=\left[\begin{array}{ccccc} M_{\varphi \varphi } & M_{\varphi v} & M_{\varphi p} & -C_{b}^{n} & 0_{3\times 3}\\ M_{v\varphi } & M_{vv} & M_{vp} & 0_{3\times 3} & C_{b}^{n}\\ 0_{3\times 3} & M_{pv} & M_{pp} & 0_{3\times 3} & 0_{3\times 3}\\ 0_{3\times 3} & 0_{3\times 3} & 0_{3\times 3} & 0_{3\times 3} & 0_{3\times 3}\\ 0_{3\times 3} & 0_{3\times 3} & 0_{3\times 3} & 0_{3\times 3} & 0_{3\times 3} \end{array}\right]\left[\begin{array}{c} \varphi \\ \delta v^{n}\\ \delta p^{n}\\ \varepsilon^{b}\\ \nabla^{b} \end{array}\right]+\left[\begin{array}{cc} -C_{b}^{n} & 0_{3\times 3}\\ 0_{3\times 3} & C_{b}^{n}\\ 0_{3\times 3} & 0_{3\times 3}\\ 0_{3\times 3} & 0_{3\times 3}\\ 0_{3\times 3} & 0_{3\times 3} \end{array}\right]\left[\begin{array}{c} \eta_{g}^{b}\\ \eta_{a}^{b} \end{array}\right] \end{array}$$

In Equation (18), $\eta_{g}^{b}$ and $\eta_{a}^{b}$ denote the random noise of the gyroscope and accelerometer, respectively. The matrices F(t) and G(t), respectively, denote the state transition matrix and process noise-driving matrix, where the specific configurations of components $M_{\varphi \varphi }$, $M_{\varphi v}$, $M_{\varphi p}$, $M_{v\varphi }$, $M_{vv}$, $M_{vp}$, $M_{pv}$, and $M_{pp}$ are formally defined in [41].

(19) $$Z(t)=\left[\begin{array}{l} {\overset{\wedge }{L}}_{INS}-{\overset{\wedge }{L}}_{HSBS}\\ {\overset{\wedge }{\lambda }}_{INS}-{\overset{\wedge }{\lambda }}_{HSBS} \end{array}\right]=H(t)X(t)+v(t)$$

**Figure 12:**
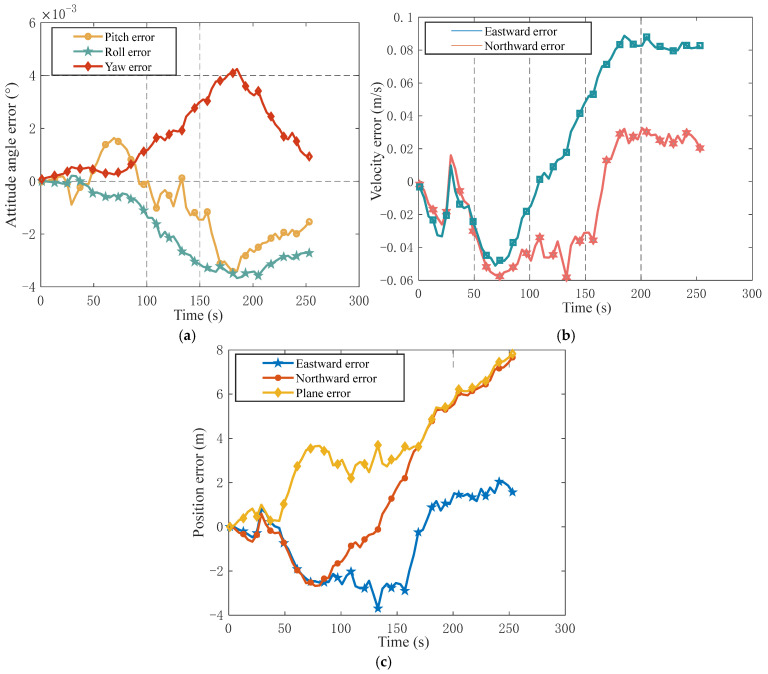
Error of integrated navigation: (**a**) attitude angle error; (**b**) velocity error; (**c**) plane error.

The measurement equation is shown in (19), where H is the system measurement matrix, v(t) is the white noise in plane position measurement, $L_{HSBS}$ and $\lambda_{HSBS}$ are the latitude and longitude information obtained by single-beacon multi-primitive method, and $L_{INS}$ and $\lambda_{INS}$ are the latitude and longitude provided by inertial navigation. The results are shown in Figure 12.

As shown in the figure above, the integrated navigation can effectively improve navigation and positioning accuracy. The accumulated positioning error is about 2 nmi and the average plane error is 4.11 m after entering the standard campus. The average east speed error is 0.038 m/s, the average north speed error is 0.063 m/s, the average pitch angle error is 9.36″, the average roll angle error is 7.92″, and the average heading angle error is 9.36″.

## 6. Conclusions

This paper focuses on the ranging conditions of an underwater acoustic beacon, aiming to explore how to effectively use the INS, Log, and PS of an AUV to achieve real-time and high-precision navigation and positioning in an underwater environment. An improved range-only single-beacon location algorithm with DCGN is proposed in this paper. This algorithm combines the Chan algorithm and the improved Gauss–Newton algorithm, and realizes the accurate determination of underwater carriers in the absence of prior position information by constructing the underwater virtual beacon positioning model. A simulation experiment and lake test show that this algorithm not only has a faster convergence speed, but also has an average positioning accuracy of 4.16 m when the positioning error of 2 nmi is accumulated, which is a reliable algorithm.

## Figures and Tables

**Table 1:** Sensor performance index.

Sensors	Parameters	Values
Gyroscope	bias stability	0.002 °/h
	angular random walk	$<0.0003\;{}^{\circ}/\sqrt{h}$
	scale factor nonlinearity	<2 ppm
	output frequency	200 Hz
Accelerometer	bias stability	<20 μg
	random walk coefficient	<5 μg
	scale factor nonlinearity	<2 ppm
	output frequency	200 Hz

## Data Availability

Data are contained within the article.
